# New Method of Impact Localization on Plate-like Structures Using Deep Learning and Wavelet Transform

**DOI:** 10.3390/s25061926

**Published:** 2025-03-20

**Authors:** Asaad Migot, Ahmed Saaudi, Victor Giurgiutiu

**Affiliations:** 1 Department of Petroleum and Gas Engineering, College of Engineering, University of Thi-Qar, Nasiriyah 64001, Iraq; 2 Department of Mechanical Engineering, University of South Carolina, 300 Main Street, Columbia, SC 29208, USA; ahmed.saaudi@mu.edu.iq (A.S.); victorg@sc.edu (V.G.); 3 Department of Communication and Electronics Engineering, College of Engineering, University of AL-Muthanna, Samawah 66001, Iraq

**Keywords:** impact localization, wavelet transform (WT), deep learning (DL), convolutional neural network (CNN), PWAS

## Abstract

This paper presents a new methodology for localizing impact events on plate-like structures using a proposed two-dimensional convolutional neural network (CNN) and received impact signals. A network of four piezoelectric wafer active sensors (PWAS) was installed on the tested plate to acquire impact signals. These signals consisted of reflection waves that provided valuable information about impact events. In this methodology, each of the received signals was divided into several equal segments. Then, a wavelet transform (WT)-based time-frequency analysis was used for processing each segment signal. The generated WT diagrams of these segments’ signals were cropped and resized using MATLAB code to be used as input image datasets to train, validate, and test the proposed CNN model. Two scenarios were adopted from PAWS transducers. First, two sensors were positioned in two corners of the plate, while, in the second scenario, four sensors were used to monitor and collect the signals. Eight datasets were collected and reshaped from these two scenarios. These datasets presented the signals of two, three, four, and five impacts. The model’s performance was evaluated using four metrics: confusion matrix, accuracy, precision, and F1 score. The proposed model demonstrated exceptional performance by accurately localizing all of the impact points of the first scenario and 99% of the second scenario. The main limitation of the proposed model is how to differentiate the data samples that have similar features. From our point of view, the similarity challenge arose from two factors: the segmentation interval and the impact distance. First, applying the segmenting procedure to the PWAS signals led to an increase in the number of data samples. The procedure segmented each PWAS signal to 30 samples with equal intervals, regardless of the features of the signal. Segmenting and transforming different PWAS signals into image-based data points led to data samples that had similar features. Second, some of the impacts had a close distance to the PWAS sensors, which resulted in similar segmented signals. Therefore, the second scenario was more challenging for the proposed model.

## 1. Introduction

The significant development in the aerospace industry has led to an increase in the interest of using airplanes as fast and comfortable vehicles. Throughout the service life of these airplanes, their structures age. These aging airplanes need reliable structural health monitoring (SHM) systems to investigate and monitor the structural integrity and prevent air accidents. The SHM system includes sensors installed on the structure to be monitored. These sensors can sense the acoustic emission signals from various sources such as a fatigue crack and an impact event of a foreign object. The SHM system will analyze the received signals to identify and localize the source of these signals. Based on the SHM report, a maintenance process can be implemented on the investigated structure using non-destructive testing (NDT) techniques [1,2]. The impact events represent one of the biggest concerns for moving structures such as aircraft. These impact events may cause visible or invisible structural damage. So, an efficient SHM system is needed to ensure structural integrity, analyze generated AE signals, and localize their sources [3]. Based on sensor functionality, The SHM systems include active and passive systems. The active SHM system excites a structure by an active sensor in a specific manner and receives measured signals from the receiver’s sensors to quantify the structural damages [4,5]. In a passive SHM system, the used sensors acquire the structural signals generated from impacts or damage initiation [6,7].

Acoustic emission (AE) represents one of the effective SHM techniques that has been used for the inspection and evaluation of damage in aerospace applications. Comprehensive studies have been implemented using the AE tool to determine impact location and resulting structural damages. This AE technique involves a network of sensors that are permanently installed over the investigated structure to acquire AE signals generated due to structural damage initiation [8,9,10]. In these traditional studies, the AE signals received by a network of sensors were analyzed to determine the time of flight (TOF) of these signals. The TOF is used in different approaches to localize the AE source. The triangulation approach based TOF is widely used for localizing the acoustic source or impact events in isotropic (metallic) and quasi-isotropic materials with a percentage of error. This approach needs the TOF of three signals received by three sensors to find the intersection point of three circles. The propagating wave group velocity is assumed to be known and constant in all directions. The story is different for anisotropic composite materials where the group velocity of propagating waves their dependent on its direction. Several methodologies have been developed to overcome this issue and localize the AE source on anisotropic composite materials [11,12,13]. The edge reflection waves that appear in the received AE signal may affect the accuracy of estimating the TOF and AE source location [14]. Several studies have taken advantage of the extra information revealed by reflection waves and reverberations to increase the accuracy of AE source localization. For, example, the AE sources were localized using three sensors, which were used to acquire guided wave reflection forms [15]. An analytical model was developed to decrease the number of sensors used and improve the accuracy of localizing AE sources on metallic plates. This model was used to simulate the reverberation forms of the AE signal. The AE waves were rebuilt by using direct (first) arrivals that were recorded experimentally [16].

Most of the presented methods for localizing AE sources are classified as model-based methods [17]. These methods need to determine specific features from the acquired data to localize the AE source, such as the time of flight (TOF). These methods do not accurately localize the AE emission sources because they are based on the sensors’ location, wave propagation velocity, and appropriate threshold value settings to estimate the TOF of acoustic signals [18].

In recent years, several kinds of data-driven studies have been implemented to localize AE sources. These approaches dispensed with the requirement to extract specific features from the acquired data. For example, the TOF of acoustic signals, the type of propagating Lamb wave modes and their central frequency values, and the direction of propagation of Lamb wave modes affected the group velocity values of received acoustic waves [19]. Identifying these features was crucial for localizing impacts. Machine learning (ML) methods, like artificial neural networks (ANNs), are a widely utilized technique for impact detection and investigation [20,21]. This type of ML can modify its weight while it is being trained. ANNs are often effective in the case of a wide variety of given training datasets [22]. Impact points on simple structures have been localized using other ML methods, such as extreme learning machines (ELMs) [23]. Sause et al. [24] developed an ANN technique based on sonic emissions to anticipate failure load and assess a material’s current stress exposure during testing. Datta et al. [25] illustrated the ability of a developed method to locate an impact event on a composite plate using X and Y coordinates and its energy using the least squares support vector regression-based ANN approach. The ANN’s requirement for extensive training sets is one of its drawbacks. To enhance the learning process, there is a need for large and diverse data samples. The requirement for size and diversity remains a significant challenge in the learning process. Data augmentation algorithms have been employed to artificially increase the number of data points based on the available samples. Techniques such as noise addition, signal scaling, and time shifting were used to generate a larger dataset. The augmented dataset was specifically utilized to train the machine learning models. The incorporation of artificial data points addressed the data size challenge faced by many research efforts. However, the use of augmentation algorithms could produce data observations with similar features. This similarity could lead to overfitted models [26].

Convolutional neural networks (CNNs) are deep learning approaches that have several domains, such as the recognition of objects, the classification of images, etc. [27,28]. These approaches have been used in many studies on structural damage detection and AE source localization because of their outstanding achievement. A novel metamodel-based CNN was proposed to localize impact points on complex composite structures using a network of PWAS sensors installed to receive generated impact waves. These waves were transferred to a set of two-dimensional images that were used as input data for the proposed CNN system to detect the impact points [29]. Ebrahimkhanlou [30] used a CNN and stacked autoencoders to localize the zones of pencil lead break tests on a stiffened metallic plate using a single sensor. The results showed that relevant information about the sources’ locations could be found in the reverberation patterns of AE sources. The impact events that were near the edges of the composite structure were localized using a CNN based on the time–frequency of received signals. The result showed that the CNN system randomly localized the impact signals in 12 zones with acceptable localization error [31]. The localization of load impact on complex structures like ships was achieved using the MSFF-CNN method. This method helped to avoid the procedure for obtaining signal features where the model receives the raw vibration signals of impact loads directly. The results showed excellent accuracy of impact load localization [32]. Khan et. al. [33] presented a CNN-based vibration analysis method for detecting delamination composite structures. Zhao et al. [34] developed a new methodology for localization impact events distributed on small areas of simple test plates. They used a network of four sensors to acquire signals of 1800 impact events distributed in 45 small areas (each area had 40 impact events). These received time–domain signals were transferred to images using wavelet transform to be used as input image datasets for the proposed CNN-based TL model. The suggested approach with transfer learning and a limited dataset was simple to apply to new composite structures, offering a novel SHM.

Most of the previous studies were based on data-driven zonal localization for AE sources or impact events. In this work, impact events on plate-like structures were classified and localized as points using the proposed CNN method based on limited received impact signals. The received signals (at least two signals received by two sensors) of each impact event could be used to validate and test the proposed CNN system. While most of previous studies prepared large datasets to validate and test an AI system. For DL-based SM evaluation, obtaining a model with good performance with less data has become a critical issue. For this, the augmentation algorithms were implemented to artificially increase the number of data samples by applying various data transformations. Several augmentation techniques were employed, including zoom range and horizontal flipping. The newly generated data samples were exclusively used during the training process to enhance the model’s generalization and performance. Although augmentation techniques are an effective solution for improving the model training process with limited data samples, excessive augmentation may lead to an overfitted model.

## 2. Experimental Setup and Results

**Figure 1:**
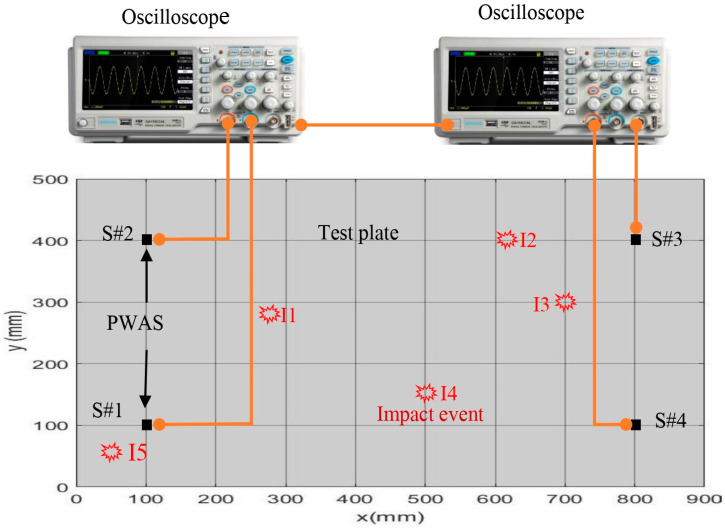
Experimental setup of recording the signals of five impact events.

Four PWAS transducers were installed on the corner of a simple aluminum plate with dimensions of 900 mm × 500 mm × 1 mm. The test plate was subjected to low-velocity impact by a small steel ball weighing 0.33 g. This ball was dropped vertically through a small pipe (50 mm in length). The generated impact signals were received by four PAWS transducers and recorded by two oscilloscopes, as shown in Figure 1. The recorded length was 92,000 points with a sampling rate of 5 M samples/s. In this work, five impact points with the dimensions illustrated in Table 1 were investigated.

**Figure 2:**
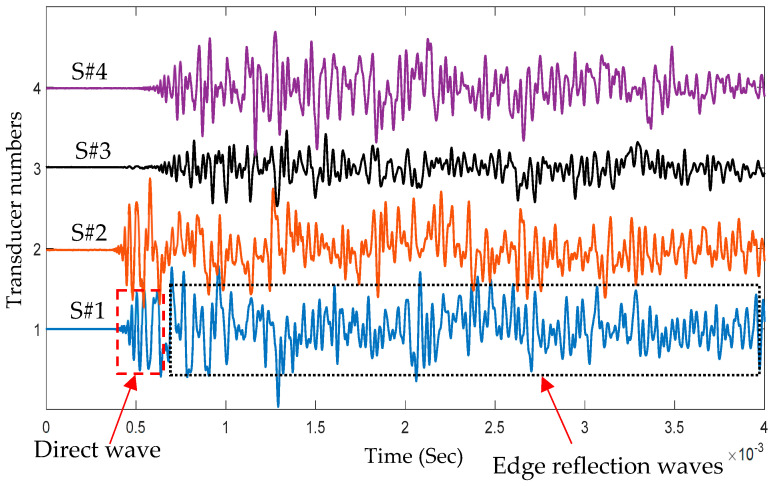
Four time–domain signals of impact event I1 received by four PWAS transducers (S#1, S#2, S#3, S#4).

**Figure 3:**
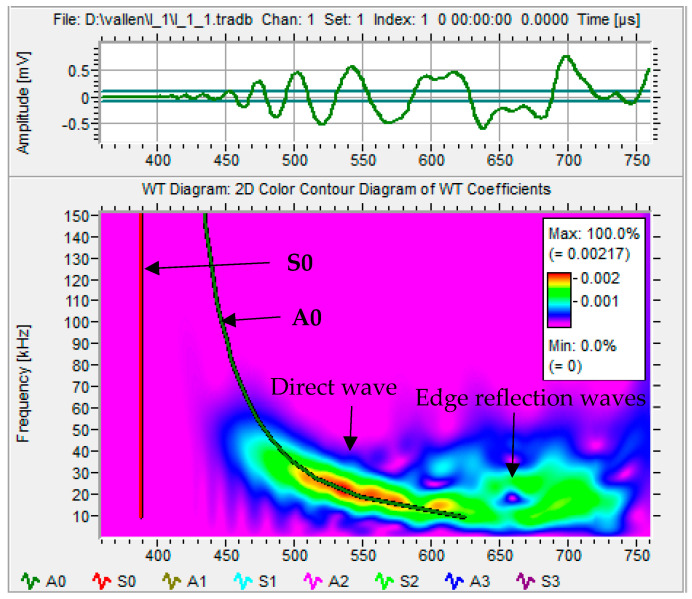
The WT of the signal received by PWAS S#1 due to impact event I1. The WT overlapped with the relevant group–velocity curves (dispersion curves) of the Lamb waves.

Figure 2 shows four impact signals of impact point I1 received by four PWAS transducers: S#1, S#2, S#3, and S#4. Each impact signal included direct and edge reflection signals. Based on the time of flight of the received signal and its energy (signal amplitude), it could be concluded that impact event I1 was located close the sensors S#1 and S#2 and far away from the sensors S#3 and S#4. The wavelet transform (WT) of an impact wave may offer a wealth of pertinent details because it indicates which modes carry considerable energy and where that energy is located within a mode as a function of frequency. Adjustable windows in wavelet transform (WT) can monitor time and frequency information more effectively [35]. The free software AGU-Vallen Wavelet (http://www.vallen.de/wavelet/index) was used to generate the WTs for impact signals [36]. The “mother” wavelet in this program is the Gabor function. The Gaussian shape of the Gabor wavelet in both the time and frequency domains allowed for a good balance between time and frequency resolution. Figure 3 shows the WT of the signal generated by impact event I1 and received by PWAS transducer S#1 and the time–frequency domain of this signal. Because of low-velocity impact events, we could observe only the antisymmetric Lamb wave mode (A0). The WT contour showed that the signal energy was concentrated in the area of anti-symmetric Lamb wave mode (A0) and there was no energy in the area of symmetric Lamb wave mode (S0), which meant that only A0 was present in the impact signals. The WT involved a direct wave and edge reflection waves. The maximum amplitude of the direct wave was at a frequency value of 20 kHz.

## 3. Preparing Input Datasets for CNN Models

**Figure 4:**
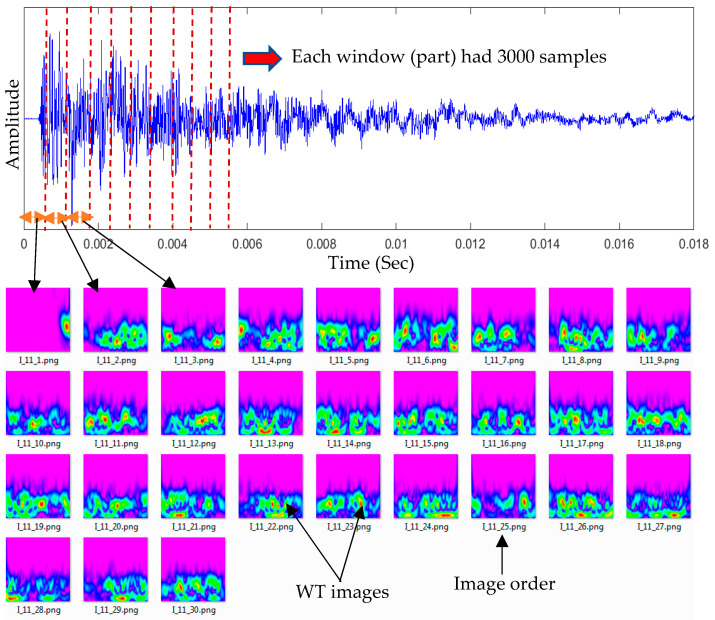
Each received time–domain signal was divided into 30 equal parts (windows). Each signal part was transformed to a time–frequency domain using wavelet transform.

The input image dataset for the proposed CNN model was prepared. Each impact event had four impact signals received by sensors S#1, S#2, S#3, and S#4. Each signal, which had 92,000 samples, was divided into 30 parts (each part had 3000 samples). The WT was generated for each part of the signal, as shown in Figure 4. The first image represented the WT of the direct wave (incident wave). The other 29 images included the WT of the reflection waves. These WTs had useful details about the impact event. The input image dataset for the proposed CNN model was conducted using a prepared MATLAB R2016a code. This code was used to customize, crop, and resize the images to 224 × 224 pixels. The 120 images were prepared for each impact event and its generated waves were received by four sensors (30 images for each received signal).

### Data Augmentation

The WT signals in both the two-sensor and four-sensor scenarios were collected and processed to generate eight image-based datasets, representing two, three, four, and five impacts. In the two-sensor scenario, the number of data samples per impact was 60, while, in the four-sensor scenario, it was 120. To enhance the model performance, data augmentation techniques such as rescale, shear range, zoom range, and horizontal flip were employed to increase the number of data samples during the training of the models.

## 4. Impact Event Localization and Classification Using the CNN

**Figure 5:**
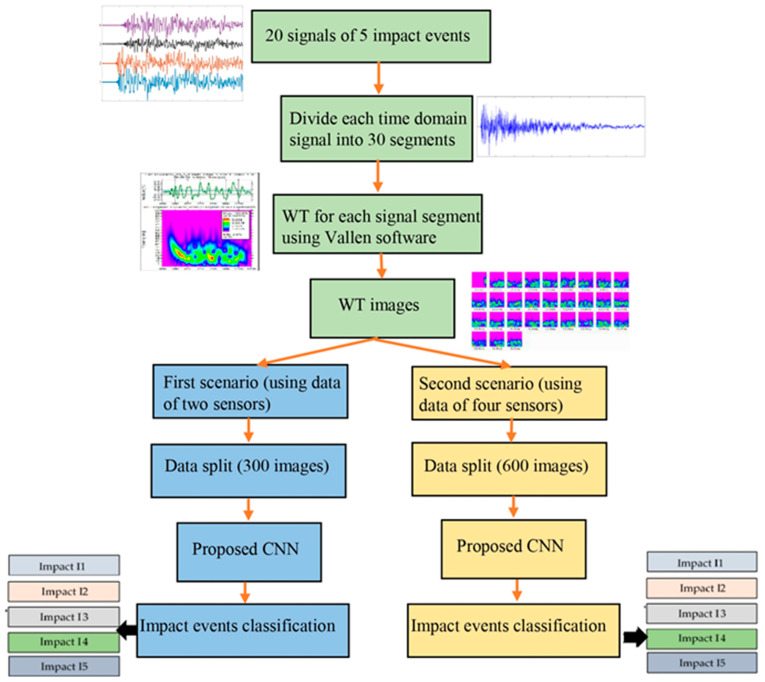
The schematic shows the workflow of the proposed CNN model.

As stated in Section 3, an image dataset for each impact event was prepared. In this work, two scenarios were taken into consideration to classify the impact events. First, two sensors (S#1, S#3) were used to localize the impact events. Each impact event had 60 WT images. The CNN model included four separate processes to classify two impact events, three impact events, four impact events, and five impact events. Second, four sensors (S#1, S#2, S#3, S#4) were used to localize the impact events. Each impact event had 120 WT images. The CNN model repeated the same classifying processes with an image dataset twice the size of that in the first scenario. Figure 5 shows a schematic of how to use the suggested CNN model to classify impact events. The image datasets were randomly divided into 70% training dataset, 10% validating dataset, and 20% testing dataset. For example, the image dataset for classifying five impact events using two sensors (first scenario) had 300 images (each impact event had 60 images). The training dataset had 210 images, the validating dataset had 30 images, and the testing dataset had 60 images.

### 4.1. Designed CNN Model

**Figure 6:**
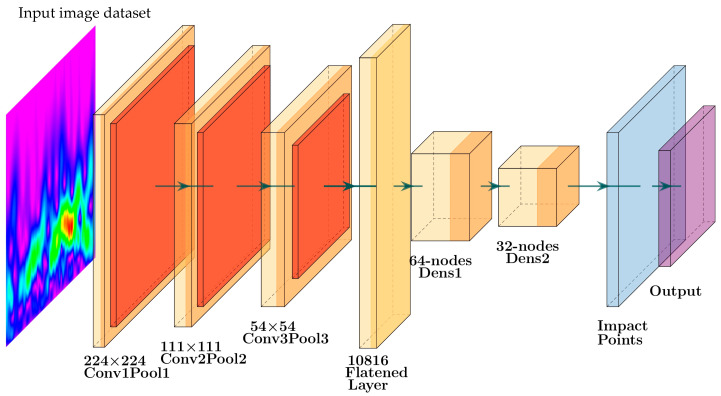
The framework of the proposed CNN model.

The proposed model consists of an input layer, three two-dimensional CNN layers with their corresponding max-pooling layers, three dense layers, and an output layer (see Figure 6). The input layer receives a sample of an image-based PWAS signal of 224 × 224 pixels. Then, the first CNN layer applies 64 filters of size 3 × 3 with a stride of size 1 to produce 64 feature maps of 222 × 222. Applying a 3 × 3 filter with a single stride and no padding reduces the dimensions to 222 × 222. Within the receptive field of a 3 × 3 filter, the convolution operation is applied to identify the main local features of the signal by calculating the local weighted sum (see Equation (1)). The convolution operation is set to use the Rectified Linear Unit (ReLU) activation function, as shown in Equation (2). The weighted sum of each local receptive field is passed to ReLU. Then, the activation function passes the positive features that have meaning and suppresses the negative ones. Next, the max-pooling layer of window size 2 × 2 is applied to reduce the dimension to 111 × 111 × 64. The max-pooling keeps the strongest activated feature within a 2 × 2 window; see Equation (3) for the mathematical representation. Then, the second CNN layer uses 32 filters of size 3 × 3 on 111 × 111 maps to produce 109 × 109 × 32 feature maps. Next, a max-pooling layer of size 2 × 2 is used to reduce the complexity to 54 × 54 × 32. After the second CNN layer, a drop-out layer is added with a drop rate (0.3). This layer generalizes the behavior of the model by dropping 30% of the weights randomly during training. This weight-dropping removes or reduces the overfitting that could have happened during the model training. Then, a third CNN layer of 16 filters with its corresponding max-pooling layer is added. The result is 16 feature maps of size 26 × 26. Next, these feature maps are flattened into 10,816 nodes. Then, two dense layers are added with 64 and 32 nodes, correspondingly. The final stage is the output layer. The number of nodes in the output layers varies from 2 to 5 according to the number of impact points. The softmax activation function is used with the output layer to predict the impact points.

(1) $$Z\left(i,j\right)=\sum_{m=0}^{2}\sum_{n=0}^{2}X\left(i+m,\;j+n\right)\cdot W\left(m,\;n\right)+b$$

where *X* is the input feature map. *W* is a filter kernel of size 3 × 3. *b* is the bias term (scalar)*. Z* (*i*, *j*) is the output feature map value at position (*i*, *j*). The filters move over the input image-based signal with a stride of 1.

(2) $$f\left(x\right)=\left\{\begin{array}{c} x,\;if\;x>0\\ 0,\;if\;x\leq 0 \end{array}\right.$$

where *x* is the weighted sum of inputs from the previous layer (*Z*).

(3) $$P\left(i,j\right)=max\left\{X\left(2i,2j\right),X\left(2i,2j+1\right),X\left(2i+1,2j\right),X\left(2i+1,2j+1\right)\right\}$$

where *P* (*i*, *j*) is the output of the pooled feature map at position (*i*, *j*). *X* (m, *n*) is the input feature map value at position (*m*, *n*). The indices (2*i*, 2*j*) define the top-left corner of the 2 × 2 pooling window.

The optimization algorithm Adam and the loss function (sparse categorical cross entropy) were used in the training procedure. The utilized learning rate for Adam was 0.001. The number of training epochs was set to 300 iterations. In addition, the use of sparse categorical cross-entropy loss function did not need to hot encode the labels of the impact points. Instead, the sparse categorical cross entropy worked with the labels that were integer encoded; for a mathematical representation, see Equation (4).

(4) $$Loss=\frac{1}{N}\sum_{i=1}^{N}Log\left({y^{\prime }}_{i,true}\right)$$

where *N* is the number of samples in the batch. ${y^{\prime }}_{i,true}$ is the predicted probability for the class true for the *i* sample. This value comes from the network’s softmax output. The *log* is a natural logarithm.

The proposed CNN-based architecture involves 719,381 trainable parameters. Table 2 illustrates the proposed structure by presenting each layer with its output shape and trainable parameters. For instance, for the first two-dimensional convolutional layer (conv2d), an input data sample of size 222 × 222 is convoluted with 64 filters to produce 64 feature maps. This process consists of 1792 trainable parameters. The proposed model is characterized by clarity and a moderate number of trainable parameters.

In this work, the model behavior was examined on the identification of two, three, four, and five impacts. For this, the model was trained with four different datasets, and the structure of the output layer was changed to 2, 3, 4, or 5 nodes.

### 4.2. Evaluation of Model Performance

In this work, four metrics [37,38] were utilized to evaluate model performance: precision, recall, F1 score, and accuracy. This section provides a theoretical analysis of these evaluation metrics. The accuracy metric assessed the overall performance of identifying region points without considering the distribution of points. Equation (5) presents the mathematical calculation of accuracy, which was determined by dividing the number of correct predictions by the total number of predictions.

(5) $$\mathrm{Accuracy}=\frac{TP+TN}{TP+TN+FP+FN}$$

The precision metric was used to determine the percentage of positive samples that were correctly predicted as True Positives, relative to the total number of samples predicted as positive (see Equation (6)). Additionally, the recall metric measured the proportion of positive samples that were correctly predicted compared to the total number of actual positive samples (see Equation (7)). Finally, the F1 score was employed to represent the balance between precision and recall. The primary concept of the F1 score was to weigh these two ratios and present their harmonic mean, as illustrated in Equation (8).

(6) $$\mathrm{Precision}=\frac{TP}{TP+FP}$$

(7) $$\mathrm{Recall}=\frac{TP}{TP+FN}$$

(8) $$F1\;\mathrm{score}=\frac{2\times Precision\times Recall}{Precision+Recall}$$

### 4.3. Results and Discussion of Impact Identification Models Using Two Sensors

**Figure 7:**
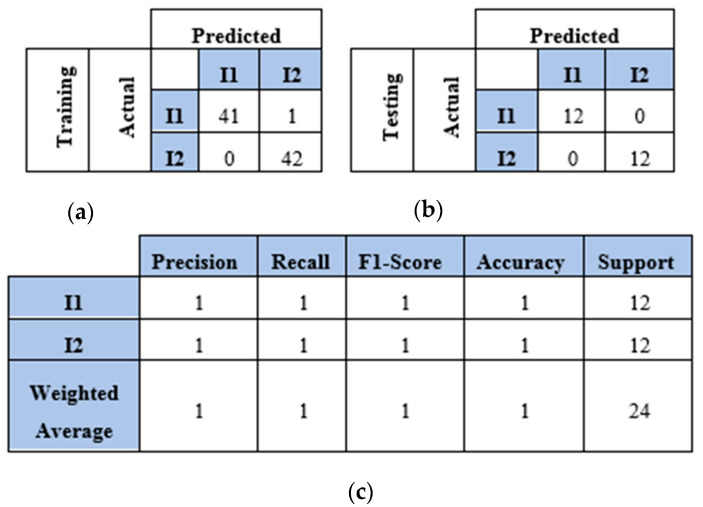
The CNN results for identifying two impact events (I1 and I2) when signals were received by two sensors (S#1 and S#3): (**a**) confusion matrix of training dataset; (**b**) confusion matrix of testing dataset; (**c**) classification report. Each impact event had 60 images prepared from two signals.

**Figure 8:**
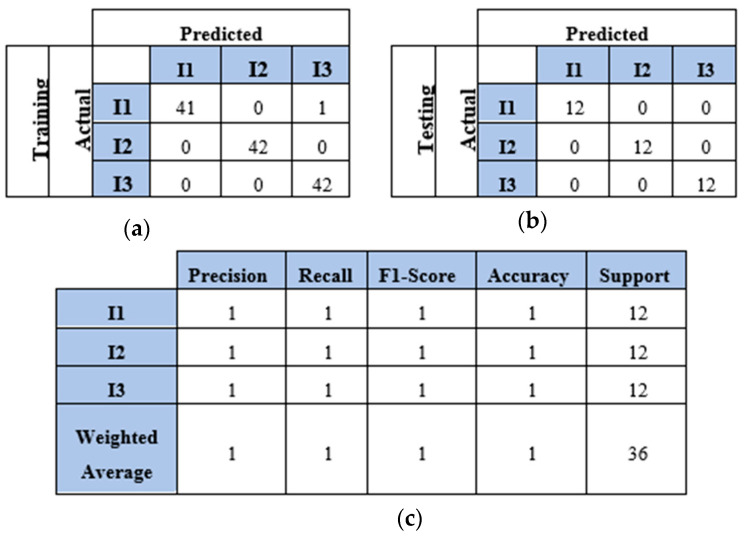
The CNN results for identifying three impact events when signals were received by two sensors (S#1 and S#3): (**a**) confusion matrix of training dataset; (**b**) confusion matrix of testing dataset; (**c**) classification report. Each impact event had 60 images prepared from two signals.

**Figure 9:**
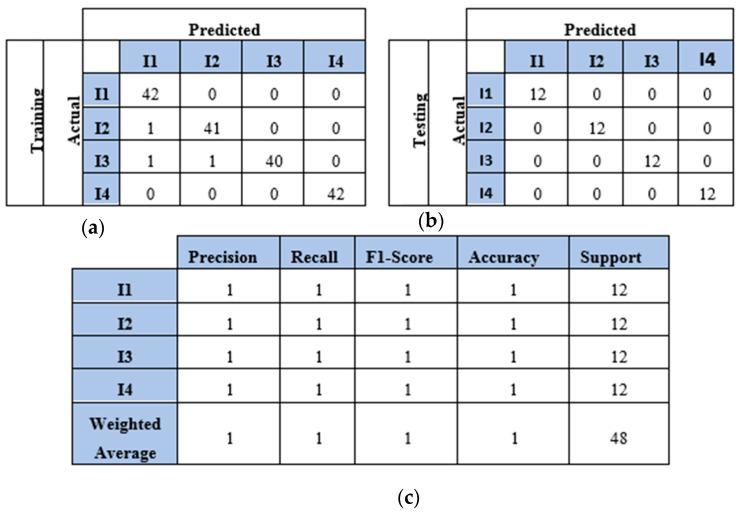
The CNN results for identifying four impact events when signals were received by two sensors (S#1 and S#3): (**a**) confusion matrix of training dataset; (**b**) confusion matrix of testing dataset; (**c**) classification report. Each impact event had 60 images prepared from two signals.

**Figure 10:**
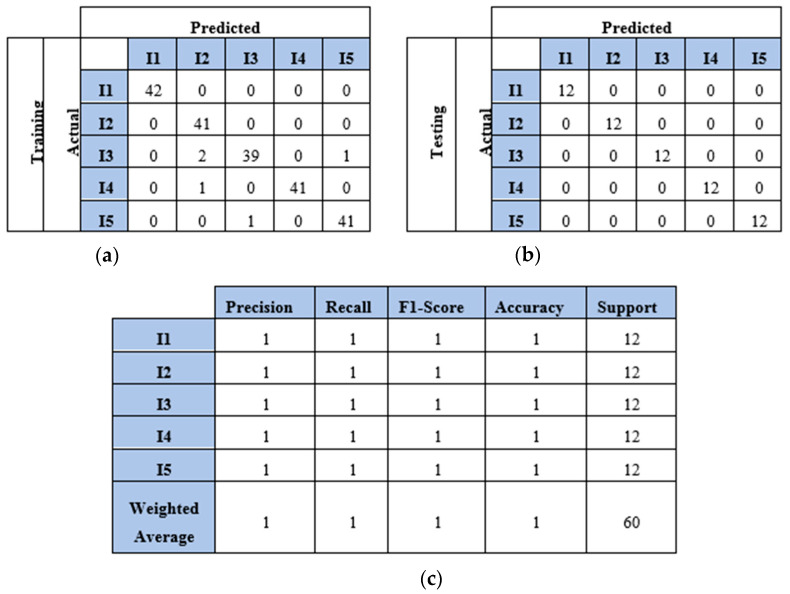
The CNN results for identifying five impact events when signals were received by two sensors (S#1 and S#3): (**a**) confusion matrix of training dataset; (**b**) confusion matrix of testing dataset; (**c**) classification report. Each impact event had 60 images generated from two signals.

**Figure 11:**
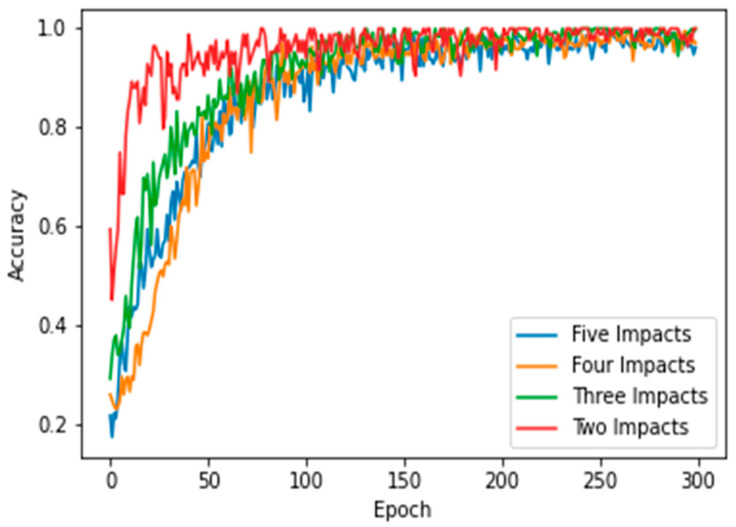
Learning curves of the proposed CNN model for classifying 2, 3, 4, and 5 impact events. Two sensors (S#1 and S#3) were used to receive the impact events signal. Each impact event had 60 images generated from two signals.

This section presents the results of the first scenario, where a two-sensor setup was adopted. The proposed model was trained and tested using two, three, four, and five impact datasets. The following subsections present the evaluation results using the confusion matrix, precision, recall, and F1 score for each dataset. First, the confusion matrix in Figure 7 shows the model’s behavior for impact I1 and impact I2. The confusion matrix of the model with the training dataset is illustrated in Figure 7a. The model falsely predicted one point of impact I2. Moreover, the model predicted all of the testing samples correctly, as shown in Figure 7b. Based on the confusion matrix of the testing dataset, the classification is presented to demonstrate the model’s behavior by monitoring the distribution of data samples. Four metrics were used to evaluate the model’s precision, recall, F1 score, and accuracy. All the metrics showed the high performance of the model (see Figure 7c). Second, with three impacts, the model classified all the regions correctly, as shown in Figure 8a,b. Both training and testing examples were identified correctly (see the classification report, Figure 8c). Third, the model truly identified all the image-based testing samples of four impacts, as shown in Figure 9b,c. The evaluation metrics illustrate that the model was accurate for impacts using the testing data samples. However, the model falsely predicted three samples of the training dataset (see Figure 9a); one sample of impact I2 was identified as impact I1 and two samples of impact I3 were predicted as impact I1 and I2. Finally, with the five impact datasets, the model accurately recognized all the data samples from the testing dataset, as illustrated in Figure 10b,c. However, the model incorrectly predicted six samples from the training dataset, as shown in Figure 10a. To monitor the model’s learning progress over epochs, Figure 11 presents the learning curves for the model trained on two, three, four, and five impact datasets. The curves illustrate the model’s convergence over time from 1 to 300 epochs. During the first 50 epochs, the model struggled to identify the WT samples. However, the model began to converge as it learned additional features from the data samples.

### 4.4. Results and Discussion of Impact Identification Model Using Four Sensors

**Figure 12:**
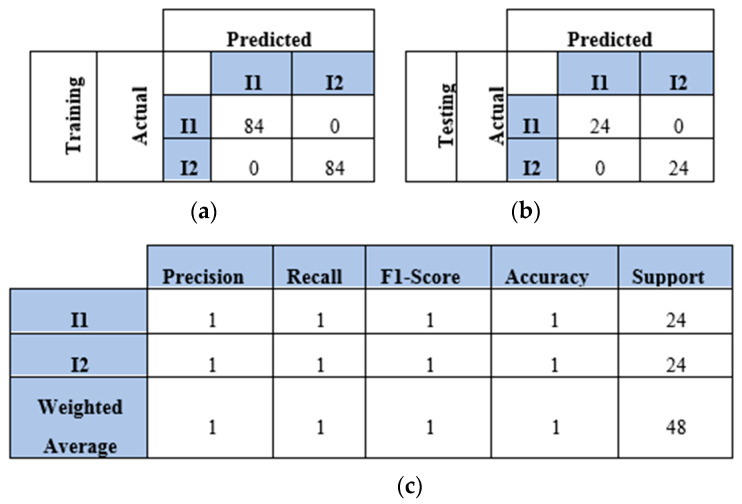
The CNN results for identifying two impact events when signals were received by four sensors (S#1, S#2, S#3, and S#4): (**a**) confusion matrix of training dataset; (**b**) confusion matrix of testing dataset; (**c**) classification report. Each impact event had 120 images prepared from four signals.

This section presents the findings of using a four-sensor setup, where four sensors were placed to collect and process four WT datasets, as mentioned in Section 4.2. These datasets represented the WT image-based samples of two, three, four, and five impacts conducted over a plate. The results and discussion are presented in terms of confusion matrix, precision, recall, F1 score, and accuracy. The confusion matrices of the two-impact dataset are illustrated in Figure 12a,b. All training and testing points were truly identified. The classification report of the two-impact data samples is presented in Figure 12c, where scores of ones are recorded for precision, recall, F1 score, and accuracy.

**Figure 13:**
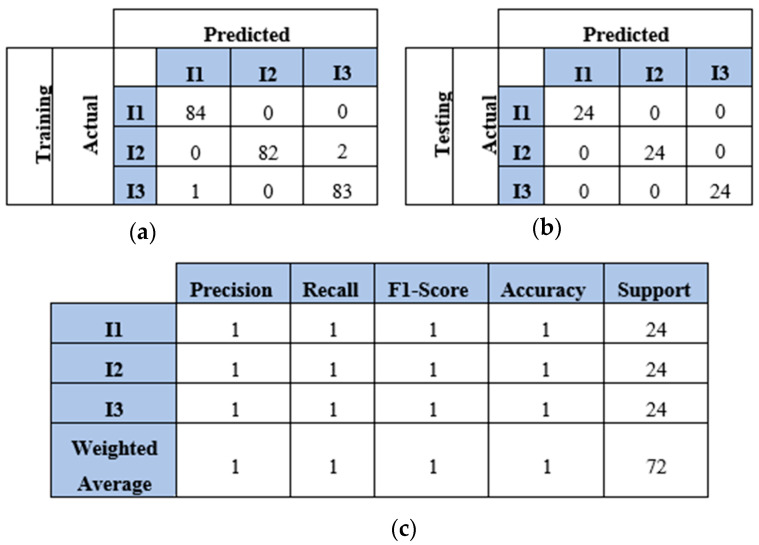
The CNN results for identifying three impact events when signals were received by four sensors (S#1, S#2, S#3, and S#4): (**a**) confusion matrix of training dataset; (**b**) confusion matrix of testing dataset; (**c**) classification report. Each impact event had 120 images prepared from four signals.

**Figure 14:**
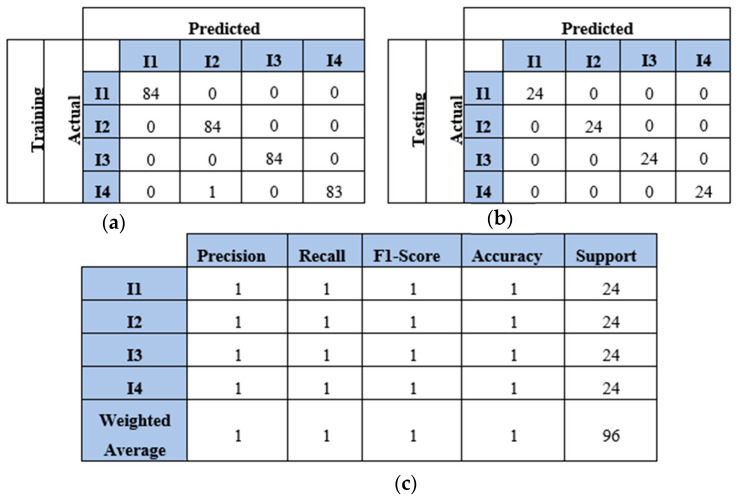
The CNN results for identifying four impact events when signals were received by four sensors (S#1, S#2, S#3, and S#4): (**a**) confusion matrix of training dataset; (**b**) confusion matrix of testing dataset; (**c**) classification report. Each impact event had 120 images prepared from four signals.

The model also predicted all the testing samples of the three-impact four-sensor dataset. Figure 13 illustrates the model’s performance with both the training and testing datasets. The model incorrectly predicted two points of impact two as impact three and one point of impact three as impact one. However, it accurately predicted all points in the testing set. The model demonstrated high accuracy with the four-impact dataset, as shown in Figure 14. The confusion matrices for both the training and testing datasets reflect the model’s performance. All points were predicted correctly, except for one point from the training set. Furthermore, the classification report presents high scores for the evaluation metrics based on the testing dataset.

**Figure 15:**
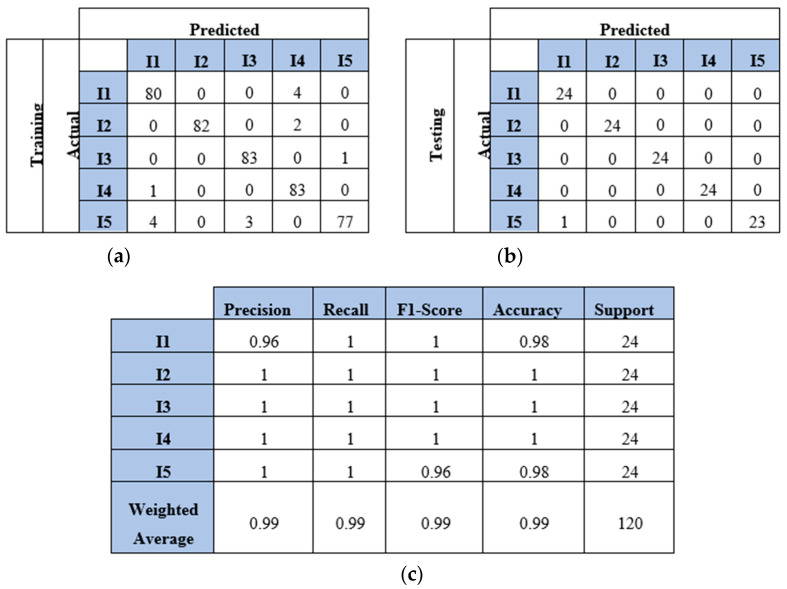
The CNN results for identifying five impact events when signals were received by four sensors (S#1, S#2, S#3, and S#4): (**a**) confusion matrix of training dataset; (**b**) confusion matrix of testing dataset; (**c**) classification report. Each impact event had 120 images prepared from four signals.

**Figure 16:**
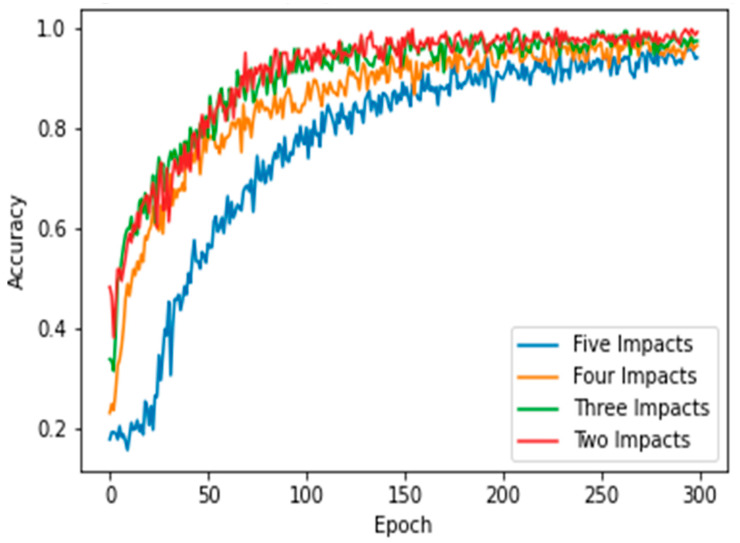
Learning curves of the proposed CNN model when classifying 2, 3, 4, and 5 impact events. Four sensors were used to receive the impact event signals. Each impact event had 120 images generated from four signals (each signal had 30 images).

Finally, the model demonstrated strong performance with the five-impact dataset in a four-sensor scenario. Figure 15a,b illustrate the model’s behavior for both the training and testing sets. Overall, the model misclassified 15 points from the training dataset and 1 point from the testing dataset. The evaluation metrics for the testing dataset are presented in Figure 15c. The average accuracy was 99%, as one instance of impact five was misclassified as impact one, as shown in Equation (5), which calculated the percentage as 119 divided by 120. Furthermore, the precision for impact one was 96%, as detailed in Equation (6). The number of true positives for impact one was 24, while the sum of true positives and false positives for impact one was 25 (24 + 1). Thus, the precision for impact one was 96%. Conversely, the recall for impact five was also 96%, as indicated in Equation (7). The number of true positives for impact five was 23, and the sum of true positives and false negatives for impact five was 24 (23 + 1). Figure 16 illustrates the learning curves for the four-sensor scenario. The learning curves of the proposed model, based on four datasets, are presented. These datasets included two, three, four, and five impact data points. Initially, the model demonstrated slower learning compared to the learning curves of the two-sensor scenario shown in Figure 11. This may be attributed to the scattered signals collected by the four sensors. Subsequently, the model began to learn more features from the five impacts and converged over time.

## 5. Conclusions and Future Work

### 5.1. Conclusions

This study successfully developed a novel methodology for localizing impact events on plate-like structures using a convolutional neural network (CNN) and impact signals acquired from a network of PAWS sensors. The signals were processed and transformed into a time–frequency domain using wavelet transform, enabling the CNN to learn from the reshaped image-based dataset. In general, the CNN layers extracted local features from the input image-based signals. This extraction process relied on a specified number of filters, such as 64, which performed convolution operations within their receptive fields to identify the necessary features. In the proposed model, these features are utilized to localize impact points. To improve the model’s performance, a substantial number of data samples was required for training to achieve convergence. However, collecting such data samples posed challenges due to the complexity of the setup conditions. To address this issue, augmentation algorithms were implemented to artificially increase the number of data samples by applying various data transformations. In this work, several augmentation techniques were employed, including zoom range and horizontal flipping. The newly generated data samples were exclusively used during the training process to enhance the model’s generalization and performance. The model’s effectiveness was tested in two scenarios involving different sensor configurations, and four distinct datasets were utilized to evaluate its performance. The results, assessed using confusion matrix, accuracy, precision, and F1 score metrics, demonstrated that the proposed model accurately localized the majority of impact events. These findings indicate that the proposed approach offers a promising solution for effective impact localization on plate-like structures, which has potential applications in structural health monitoring. The limitations of the proposed model arose in the second scenario, specifically with dataset four, which involved five impacts, yielding precision and recall values of 0.96 and 0.98, respectively.

### 5.2. Future Work

Future work will focus on optimizing the model and exploring its application to other structural forms and sensor configurations. The proposed CNN method will be used with both active and passive SHM systems to detect and quantify induced damages due to impacts. In this future work, a network of piezoelectric wafer active sensors (PWASs) will be installed on a test plate. First, the PWASs will work as passive sensors to acquire the generated high-energy impact signals. Then, the proposed CNN method will be used to detect and localize the impact events. Second, the PWAS transducer will work as an active sensor by sending guided waves through the test plate and measuring the response by a network of PWAS transducers or using a scanning laser Doppler vibrometer (SLDV). Then, the proposed CNN-based measured signals will be used to quantify the induced damages. A classification algorithm will be developed to identify impact points when multiple types of impact events occur simultaneously.

## Figures and Tables

**Table 1:** Locations of five impact events.

Impacts	I1	I2	I3	I4	I5
x/mm	275	625	700	500	50
y/mm	290	400	300	150	50

**Table 2:** The structure and trainable parameters for each layer of the proposed impact point identification system.

Model Layer	Output Shape	Parameters
conv2d	(None, 222, 222, 64)	1792
max_pooling2d	(None, 111, 111, 64)	0
conv2d	(None, 109, 109, 32)	18,464
max_pooling2d	(None, 54, 54, 32)	0
dropout	(None, 54, 54, 32)	0
conv2d	(None, 52, 52, 16)	4624
max_pooling2d	(None, 26, 26, 16)	0
dropout	(None, 26, 26, 16)	0
flatten	(None, 10,816)	0
dense	(None, 64)	692,288
dense	(None, 32)	2080
dense	(None, 5)	133
Trainable parameters: 719,381		

## Data Availability

Data is contained within the article.
